# Factorial validity and internal consistency of the PRAFAB questionnaire in women with stress urinary incontinence

**DOI:** 10.1186/1471-2490-8-1

**Published:** 2008-01-24

**Authors:** Erik JM Hendriks, Arnold TM Bernards, J Bart Staal, Henrica CW de Vet, Rob A de Bie

**Affiliations:** 1 Department of Epidemiology and Centre for Evidence Based Physiotherapy & Clinical Guidelines (CEBP), Maastricht University, Maastricht, The Netherlands; 2 Department of Research and Development, Dutch Institute for Allied Health Care (NPi), Amersfoort, The Netherlands; 3 Caphri Research Institute, Maastricht University, The Netherlands; 4 EMGO Institute, VU University Medical Center, Amsterdam, The Netherlands

## Abstract

**Background:**

To investigate the factor structure, dimensionality and construct validity of the (5-item) PRAFAB questionnaire score in women with stress urinary incontinence (stress UI).

**Methods:**

A cross validation study design was used in a cohort of 279 patients who were randomly divided into Sample A or B. Sample A was used for preliminary exploratory factor analyses with promax rotation. Sample B provided an independent sample for confirming the premeditated and proposed factor structure and item retention. Internal consistency, item-total and subscale correlations were determined to assess the dimensionality. Construct validity was assessed by comparing factor-based scale means by clinical characteristics based on known relationships.

**Results:**

Factor analyses resulted in a two-factor structure or subscales: items related to 'leakage severity' (protection, amount and frequency) and items related to its 'perceived symptom impact' or consequences of stress UI on the patient's life (adjustment and body (or self) image). The patterns of the factor loadings were fairly identical for both study samples. The two constructed subscales demonstrated adequate internal consistency with Cronbach's alphas in a range of 0.78 and 0.84 respectively. Scale scores differed by clinical characteristics according to the expectations and supported the construct validity of the scales.

**Conclusion:**

The findings suggest a two-factorial structure of the PRAFAB questionnaire. Furthermore the results confirmed the internal consistency and construct validity as demonstrated in our previous study. The best description of the factorial structure of the PRAFAB questionnaire was given by a two-factor solution, measuring the stress UI leakage severity items and the perceived symptom impact items. Future research will be necessary to replicate these findings in different settings, type of UI and non-white women and men.

## Background

Urinary incontinence (UI) is a common condition that affects approximately five to seven percent of adults in the Netherlands [1,2] increasing in the elderly to nine percent for men and 29% for women [3]. However, these estimates of prevalence are strongly influenced by the definition of UI. The most prevalent type of female UI is stress UI and it has been suggested that about 50% of patients have stress UI, 16% urgency UI and 34% symptoms of both types (mixed UI) [1,2]. UI is consistently associated with embarrassment, distress and anxiety, which may negatively affect social participation, intimate relationships and self-esteem and may have severe repercussions on the patient's quality of life (QoL) [4-6]. The relationship between UI leakage severity and perceived symptom impact is highly variable [5].

The importance of measuring the impact of UI severity on quality of life in patients is not only essential in research to assess the effectiveness of clinical interventions but also important in the assessment of outcomes in daily clinical practice. However, quality of life questionnaires are time consuming and so limited in their usage in daily clinical practice. Brief, valid and responsive outcome measures that combine UI leakage symptoms and the subjective impact on a patient's life are needed in clinical practice [7]. The PRAFAB questionnaire is potentially such an outcome measure [8] and is widely used in the Netherlands by physiotherapists [9], researchers [3,10,11] and to a lesser extent by general practitioners, urologists and gynaecologists. The PRAFAB questionnaire combines relevant objective and subjective aspects of UI severity, is quick and easy to administer and has the additional value of including the subjective aspects of UI [8,9].

Recently, the PRAFAB questionnaire has been shown to be a reliable and valid outcome measure with excellent responsiveness to clinical changes in a longitudinal study of women with stress or urgency UI [7]. In our previous psychometric study we were not able to demonstrate a two-factor solution as was theoretically expected: the more objective UI 'leakage severity' items and the more subjectively 'perceived symptom impact' items. However, the factor structure had never formally been tested before [7]. The most likely explanation is that our study was underpowered for appropriate factor analyses but, on the other hand, the PRAFAB questionnaire may also have only one strong underlying factor. Some authors recommend that adequate solutions can be obtained with five to ten participants per variable as long as there are at least 100 patients in the sample [12-15]. The data in our larger prospective cohort study allowed us to more appropriately investigate the multi dimensionality and expected two-factor structure of the PRAFAB questionnaire score in a large cohort group of women with stress UI (N = 279). Summary indices of the two subscales 'leakage severity' and its 'perceived symptom impact' may be used as separate outcomes in future research, instead of the total PRAFAB questionnaire score, thereby emphasizing that the questionnaire contains two different concepts.

The aim of this study was twofold. First, to investigate the factor structure and dimensionality of the (5-item) PRAFAB questionnaire score and secondly to replicate the construct validity of the PRAFAB questionnaire based on our previous findings.

## Methods

### Study population

Data were derived from a prognostic cohort study of 279 mainly Caucasian women (96%) with a primary or recurrent episode of stress UI referred for physiotherapy treatment by GPs or urogynaecologists. Stress UI was defined as involuntary leakage during effort, exertion or sneezing and/or coughing [16]. The 34 participating physiotherapists are experienced in women's health and work in different private practices in the Netherlands. The cohort of patients treated by these physiotherapists was followed from their initial visit up to 12 weeks (clinical course) or end of treatment.

The data consist of women who were at least 18 years of age who had had stress UI episodes for six months or more. All patients referred with a diagnosis of stress or dominant stress UI were included. In cases of uncertainty, patients received a self-evaluation 4-day diary at baseline, including the weekend, to classify whether they had either stress or predominant stress UI [17,18]. Exclusion criteria were urgency or dominant urgency UI, current pregnancy or being within 6 months after delivery, post-operative status within 6 months, patients who exhibit signs of active urinary tract infection, serious neurological pathology, diabetic neuropathy, congenital urological disorders, cognitive impairments or bladder cancer, and those who were unable to read and understand Dutch.

The characteristics of the study population are summarized in Table 1. There were no significant baseline differences between the samples with the exception of the number of patients with previous stress UI related surgery (p = 0.02). There were no missing data on baseline PRAFAB questionnaires. The medical ethics committees of the Deventer Ziekenhuizen approved the informed consent procedure and protocol. Only those participants who signed an informed consent form were included in this study.

**Table 1:** Baseline demographic and clinical characteristics: % (no.) unless otherwise stated

	Sample A (N = 140)		Sample B (N = 139)		P-value
	%	n	%	n	
Urogynaecologist referral for physiotherapy	45.7	64	46.7	65	0.94
Type UI (stress UI)	87.9	123	84.2	117	0.38
Age (year) (mean; SD)	46.8(8.2)		48.4 (8.2)		0.11
<45	36.4	51	31.7	44	
45–54	45.7	64	45.3	63	0.15
>54	17.9	25	23.0	32	
Educational level (low)	25.0	35	25.0	35	0.96
Parity (mean number; SD)	1.74 (1.3)		1.58 (1.4)		0.31
0	34.3	48	28.6	36	
1–2	43.6	61	53.6	71	0.27
≥ 3	22.1	31	17.8	32	
Onset UI symptoms (year) (mean; SD)	5.4 (7.0)		6.7 (7.9)		0.17
<1	32.1	45	36.0	50	
1–5	35.7	50	37.4	52	0.33
>5	32.1	45	26.6	37	
Menopausal status (pre [vs. post])	61.4	86	64.0	89	0.66
Physical health (poor)	23.6	33	17.3	24	0.19
Body Mass Index (> 30 kg/m^2^)	9.4	13	9.3	13	0.98
Previous UI surgery (> 6 months ago) ^a^	20.1	28	32.1	45	**0.02**
Co morbidity (yes) ^b^	24.5	34	27.9	39	0.52
Low back pain (>12 weeks) ^c^	12.9	18	10.1	14	0.44

^a ^Surgical procedures were related to women's stress urinary incontinence.

^b ^Co morbidity included (n Sample A/B): COPD (19/12), cardiovascular problems (11/14), and diabetes type-2 (6/9).

^c ^Low back pain (including those with leg pain below the knee (n Sample A/B (6/5))

### PRAFAB questionnaire

The PRAFAB questionnaire combines important objective and subjective aspects of UI severity: *Pr*otection (the use of pads), *A*mount of urine loss, *F*requency of UI, *A*djustment of behaviour due to the symptoms, and *B*ody (or self) image as a result of the stress UI symptoms (Table 2) [8]. Each item can be awarded up to 4 points (1–4) with a total PRAFAB questionnaire score of 20 points (min-max = 5–20; range = 16 points). The author(s) reported high crude agreement between assessors on total scores and 'severe' urine loss (score ≥ 14 points) compared with the Incotest [8]. The PRAFAB questionnaire discriminates between less or more than 2 gram urine loss per hour with a score ≥ 14 points. The positive predictive value of 'severe' urine loss was 83% for urine loss (> 2 gram/hour on a pad-test with standardized bladder volume). The negative predictive value was 61%. The PRAFAB questionnaire score is quick and easy to administer compared to a pad-test with standardized bladder volume or QoL questionnaires, and has the additional value of including the perceived symptom impact of UI [8,9]. In our previous psychometric study the PRAFAB questionnaire demonstrated high internal consistency (alpha = 0.82), test-retest reliability (ICC_agreement _= 0.96) and responsiveness on the total score with a minimally important change (MIC) of -3.0 and -5.0 points as a rule-of-thumb for patients classified as non-severe or severe [7]. The PRAFAB questionnaire was administered at baseline, 12 weeks of follow-up and at the end of treatment.

**Table 2:** The PRAFAB questionnaire score*

**Protection**
1. I never use protection for urine loss
2. I sometimes use protection, or I have to change my underwear because of urine loss
3. I normally use protection, or change my underwear several times a day because of urine loss
4. I always have to use protection because of urinary incontinence

**Amount**
1. The amount of urine loss is just a drop or less
2. Sometimes I loose a trickle
3. The loss of urine is so much that it wets noticeably my protection or clothes
4. The loss of urine is so much that my protection is soaked or leaks

**Frequency**
**Involuntary loss of urine occurs:**
1. Once a week or less
2. More than once but less than three times a week
3. More than three times a week, but not every day
4. Every day

**Adjustment**
**Implications of urine loss:**
1. I am not hampered in my daily activities
2. I have stopped some activities, such as some sports and physically demanding activities
3. I have stopped most physical activities that caused involuntary loss of urine
4. I almost never go out

**Body (or self) image**
1. I am not bothered by my urine loss
2. I think urine loss is annoying and troublesome, but I am not greatly bothered by it
3. Urine loss makes me feel dirty
4. I am disgusted by myself because of my urinary incontinence

**Total score:**

* The PRAFAB questionnaire is validated in Dutch. Psychometric testing of the English version is not performed. Nevertheless this questionnaire is provided in English to give the readers insight in the items and scoring system (min-max = 5–20 points; range 16 points)

### Factor analyses

A cross validation study design was used in which the total cohort of 279 patients was randomly divided into two samples, stratified for stress UI severity (29% of the patients scored ≥ 14 points on the total PRAFAB questionnaire score), in preparation for the exploratory and confirmatory factor analyses. In Sample A, comprising 140 women, exploratory factor analyses were applied to extract the number of meaningful factors or items. The factor structure of the 5-item PRAFAB questionnaire was investigated by principal component analysis (PCA).

Sample B provided an independent sample for confirmatory factor analysis (CFA) to test whether the data fit the premeditated factor solution as obtained in Sample A [12-15,19]. We used two different methods for CFA: firstly, a 'simple structure' PCA as a method of replicating the results ('simple structure' analysis) [13] and secondly, the maximum likelihood analysis [13-15,19]. A 'simple structure' replication analysis was carried out accordingly on the analysis in Sample A, based on the grounds that if 'simple structure' replication in Sample B reflects the same underlying dimensions it will be the most parsimonious explanation of the data. Moreover, if the replication analysis in Sample B demonstrates a different structure compared to those in Sample A the premeditated factor solution will be refuted [13].

Factors that emerged from the 'simple structure' PCA were rotated using promax (oblique) rotation to facilitate interpretation [12-15,19]. Item loadings ≥ 0.40 on one factor and cross loadings ≤ 0.30 on any other factor were accepted [12-15,19]. Factor loadings after oblique rotation in the maximum likelihood analysis may fall outside the range of -1.0 to +1.0. The final models were evaluated using the following fit indices: (1) chi-square goodness-of-fit test, which evaluates the significance of unexplained covariances (or covariances among measured variables that are not accounted for by the model), in which the p-values should not be significant and (2) chi-square/df ratio in which values should be < 2.0 [13-15,19].

Determination of the number of factors to be retained in the final solution was based on the following indices. Firstly, the Scree test was examined to identify the number of factors and to interpret the factor solutions [13-15,19]. Using the Scree plot we looked for a break between factors with relatively large eigenvalues (>1.0) and those with smaller eigenvalues. Factors that appeared before the horizontal break were assumed to be meaningful. Secondly, we examined the magnitude of the eigenvalues, the percentage of explained variance and factor loadings after PCA with promax (oblique) rotation. Final conclusions were based on the results of the CFA because of the more powerful test properties of factorial validity compared to the exploratory approaches [13-15,19,20].

### Internal consistency

Data from Sample B were used to evaluate construct validity and compared to Sample A. Internal consistency was assessed using Cronbach's alpha, Spearman inter-item and item-total correlations [21,22] of the different subscales or otherwise the total scale. All the items should assess different aspects of the same construct. Adequate alpha values should be higher than 0.70 and values higher than 0.80 are considered as excellent [14,23]. However, alpha values higher than 0.90 of individual items may indicate redundancy [24]. Adequate levels of item-total (item-to-scale) coefficients are scores between 0.3 and 0.9 [24]. Items with an item corrected-total correlation of less than 0.20 are likely to be assessing a different construct from the other items of that measure [24,25]. Inter-factor correlations were calculated to give further insight into the interpretability of the constructed factors as separate scales. Correlations of less than 0.70 support the multidimensionality of the questionnaire.

### Construct validity

Data from the total sample (N = 279) were used to evaluate construct validity. Construct validity examines the scale scores based on known groups or relates to other measures based on theoretically derived hypotheses concerning the concept being measured. The ability of the PRAFAB questionnaire to discriminate in UI severity between individuals was explored on baseline characteristics as was demonstrated in our previous study [7] and for example by Fultz et al. [4], Gasquet et al. [5] and Melville et al. [6]. Higher total PRAFAB scores, specifically on the urinary leakage items (amount and frequency) were expected (I) in patients referred by urogynaecologists compared to those referred by GPs [4,7], (II) in patients with failed stress UI surgery [6,7], (III) in patients with co-morbid conditions compared to those who have not [6,7], (IV) in patients with chronic low back pain (> 12 weeks) [26], (V) in patients with poor (self-rated) physical health [4,7] and (VI) in patients with a higher Body Mass Index (BMI > 30 kg/m^2^) [6,7]. Furthermore we expected lower total and subscale scores in patients with a higher education (VII) [4,7]. The results on potential factors (subscales) will be explored.

### Statistical analyses

First, we tested whether the data showed an approximately normal distribution (values of skewness and kurtosis between +1 and -1). All statistical tests were two-tailed and conducted with a type-1 error set at a P_α _< 0.05. Descriptive statistics were presented for demographic and clinical characteristics and the PRAFAB questionnaire. Summaries of categorical variables included frequency and the percentage of patients within each category. Statistical analysis comparing patient characteristics or results between groups was performed using the Student's t-test (or one-way ANOVA), or in skewed data the non-parametric chi-square or Kruskall-Wallis test where appropriate. Continuous variables were summarized with mean and standardized deviation (SD) unless otherwise noted. All analyses were performed with SPSS (version 13.0, 2005).

## Results

### Distributional characteristics of the 5-item PRAFAB questionnaire

The PRAFAB questionnaire item and total means (SD) for each sample are presented in Table 3. There were no significant differences between Sample A and Sample B. Most items were normally distributed except the item adjustment in both Samples A and B (statistical indices of skewness and kurtosis are larger than + 1 or -1).

**Table 3:** Descriptive statistics of baseline PRAFAB questionnaire scores for Sample A and Sample B^a^

	Study A (N = 140)			Study B (N = 139)			P-value
	Mean (SD)	Median	Range	Mean (SD)	Median	Range	
Protection	2.74 (0.97)	3	1–4	2.83 (0.92)	3	1–4	0.39
Amount	2.37 (0.71)	2	1–4	2.45 (0.68)	2	1–4	0.33
Frequency	2.94 (1.11)	3	1–4	2.97 (1.01)	3	1–4	0.82
Adjustment	1.44 (0.66)^b^	1	1–3	1.42 (0.64)^b^	1	1–3	0.89
Body image	2.36 (0.49)	2	2–4	2.35 (0.52)	2	2–4	0.85
Total score	11.85 (2.61)	12	7–18	12.04 (2.53)	12	6–18	0.55

^a ^No significant differences between Sample A and B (Student t-test]; verified by Kruskall-Wallis test for non parametric data).

^b ^Skewed and non normally distributions of items

### Factor analyses

#### Exploratory factor analyses in Sample A

**Figure 1:**
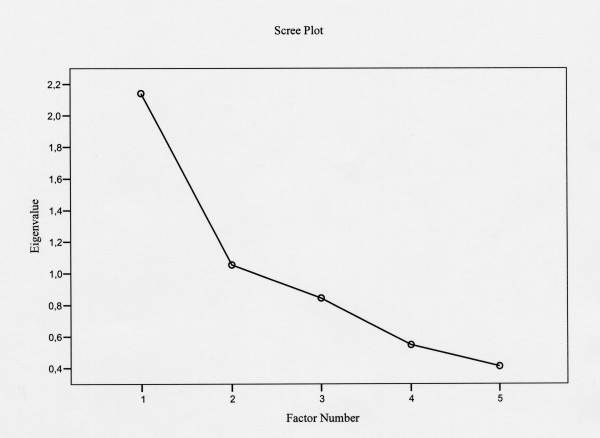
Scree plot of eigenvalues from the 5-item PRAFAB questionnaire of Sample A.

The Scree plot applied to data from Sample A showed a distinct break before factor 3, suggesting a two-factor solution of the PRAFAB questionnaire that may be adequate to describe the data (Figure 1). The first two eigenvalues of those unforced factors before rotation were 2.14 and 1.05. Note that the percentage of variance will not change following PCA but the eigenvalues will change. The eigenvalues for both factors after promax (oblique) rotation was 1.99 and 1.48 respectively. The total explained variance before rotation of the two factors was 64% of the variability of the original 5-item PRAFAB questionnaire. The initial two-factor solution accounted for 42.8% and 21.2% of the variance respectively (Table 4). The identified items with high loadings on the first factor were protection, amount and frequency (Factor 1: defined as 'leakage severity') and items with high loadings on the second factor were adjustment and body (or self) image (Factor 2: defined as 'perceived symptom impact').

**Table 4:** Results of exploratory factor analyses in Sample A followed by confirmatory factor analyses in Sample B with final factor loadings of the forced premeditated two-factor model of the PRAFAB questionnaire after promax (oblique) rotation using principal component analyses ('simple structure' replication analysis) and maximum likelihood analysis

	Exploratory Factor Analysis		Confirmatory Factor Analysis			
	Sample A (N = 140)		Sample B (N = 139)			
			Simple structure		Maximum Likelihood ^d^	
	Factor 1	Factor 2	Factor 1	Factor 2	Factor 1	Factor 2
Protection	**0.85**	0.06	**0.81**	0.23	**0.71**	0.21
Amount	**0.83**	0.23	**0.76**	0.14	**0.57**	0.13
Frequency	**0.66**	**0.40**	**0.73**	0.37	**0.58**	0.31
Adjustment	0.08	**0.79**	0.25	**0.78**	0.28	**0.40**
Body (or self) image	0.35	**0.77**	0.24	**0.84**	0.26	**0.93**
						
Eigenvalue before rotation ^a^	2.14	1.05	2.06	1.04	1.25	1.09
Explained variance (%) ^b^	42.8%	21.2%	41.2%	20.8%	25.1%	21.8%
Eigenvalue after rotation ^a,c^	1.99	1.48	1.87	1.53	1.19	1.31

^a ^Factor loadings ≥ 0.40 are in bold.

^b ^Percentage of explained variance per factor before and after oblique rotation is the same in PCA.

^c ^Because factors are correlated the rotation sum-of-squared loadings cannot be interpreted in terms of proportion of variance or added to obtain a total variance.

^d ^Chi-square goodness-of-fit test: chi-square 1.314; *df *= 1; p = 0.25

#### Confirmatory factor analyses in Sample B

In Sample B we evaluated the specified two-factor model of Sample A using a forced two-factor solution via PCA followed by promax (oblique) rotation ('simple structure' replication analysis as a CFA method). The forced two-factor solution identified the same two-factor structure as obtained in Sample A with comparable high factor loadings, confirming that these two factors may be adequate to describe the data. The results of the confirmatory analyses are shown in Table 4. The factor loadings were high, ranging from 0.73 to 0.81 for Factor 1 and 0.78 to 0.84 for Factor 2, also indicating that all the individual items are meaningful to be retained. The first two eigenvalues, as a result of the forced two-factor solution after promax (oblique) rotation using PCA, were 2.06 and 1.04. These factors accounted for 41.2% and 20.8% of the variance respectively. The first factor represented the three UI leakage items and the second factor the perceived symptom impact of stress UI.

The maximum likelihood analysis confirmed the results of the 'simple structure' analysis in Sample B although the factor loadings on the 'perceived symptom impact' subscale were slightly different compared to those of the 'simple structure' analysis. The factor loadings were considered high (> 0.40) for Factor 1 (UI leakage items), ranging from 0.57 to 0.71, and the perceived symptom impact item 'body (or self) image' (0.93) but borderline for the perceived impact item 'adjustment' with a factor loading of 0.40.

It should be noted that the frequency item in both the exploratory and confirmatory factor analysis had relatively moderate loadings or associations with Factor 2 (ranging from 0.31 to 0.40) but consistently smaller loadings in all analyses compared to the frequency items of Factor 1. In general the results of the maximum likelihood analysis were consistently lower than the results of the 'simple structure' PCA, except for the item body image which had a higher factor loading of 0.93. All individual items are meaningful to be retained although borderline for the item adjustment. The eigenvalues as a result of the maximum likelihood analysis after rotation were 1.19 and 1.31. Both factors accounted for 25.1% and 21.8% of the variance respectively. Because factors are correlated the rotation sum-of-squared loadings cannot be interpreted in terms of proportion of variance or added to obtain a total variance. The maximum likelihood analysis suggests a good model fit based on the chi-square goodness-of-fit test in which the p-value was not significant (p = 0.25) with a chi-square/df ratio of 1.31 i.e. less than the 2.0 criterion.

### Internal consistency

Internal consistencies and item-total correlations are shown in Table 5. In Sample B the Cronbach's alpha coefficient for the PRAFAB questionnaire subscale 'Leakage severity' was 0.78 with item-total correlations ranging from 0.67 to 0.85, and Spearman inter-item correlations ranging from 0.42 to 0.68, indicating that the items were rather homogenous. The Cronbach's alpha for the subscale 'Perceived symptom impact' was 0.82 with item-total correlations ranging from 0.74 to 0.84, and a slightly weaker inter-item correlation of 0.46 compared with the inter-item correlations of the subscale 'Leakage severity' (Table 5). These results were quite similar to those of Sample A. The correlation between both subscales (factors) was 0.31 in both study samples. The inter-factor correlation of less than 0.70 supports the multidimensionality of the PRAFAB questionnaire.

**Table 5:** Internal consistency and item-total correlations of the specified subscales of the PRAFAB questionnaire

	Sample A (N = 140)	Sample B (N = 139)
Internal consistency (Cronbach's alpha):		
Factor 1: leakage severity	0.82	0.78 ^a^
Factor 2: perceived impact	0.84	0.82 ^a^
Inter-factor correlations	0.31^b^	0.31 ^b^
		
Item-total correlations (range):		
Factor 1: leakage severity	0.67 – 0.83	0.67 – 0.85
Factor 2: perceived impact	0.76 – 0.87	0.74 – 0.84

^a ^Inter-item correlations ranged from 0.42 to 0.68 for Factor 1 and was 0.46 for Factor 2 in Sample B.

^b ^p < 0.01

### Construct validity

As presented in Table 6, six out of the eight hypotheses confirmed our previous findings for construct validity on baseline characteristics. Patients referred from urogynaecologists (1), having poor self-rated physical health (2), with a dissatisfying outcome following surgery (> 6 months ago) (3), with co-morbid conditions (4), or chronic low back pain (including those with leg pain below the knee) (5) had significant higher total baseline scores and on the subscale 'leakage severity'. Patients with a higher education compared to those who had not, had lower leakage severity and perceived impact scores (6).

**Table 6:** Mean total PRAFAB questionnaire and subscale scores as determined in the total group

	N (%)	Total score	Leakage severity	Perceived impact
1. Referrer				
GP	150 (53.8)	11.46 (2.39)	7.69 (1.96)	3.51 (0.65)
Urogynaecologist	129 (46.2)	12.52 (2.67) *	8.72 (2.19) *	4.10 (1.10) *
2. Type of stress UI				
Stress UI	240 (0.86)	11.85 (2.52)	8.09 (2.16)	3.69 (0.86)
Mixed UI^a^	39 (0.14)	12.49 (2.83)	8.60 (1.90)	4.30 (1.11) *
3. Education				
Low/middle	209 (74.9)	12.10 (2.54)	8.43 (2.01)	3.86 (0.98)
High	70 (25.1)	11.53 (2.62)	7.43 (1.91) *	**3.41 (0.75) #**
4. Physical health				
Poor	57 (20.4)	**12.30 (2.74) #**	8.91 (2.03) *	3.70 (0.96)
Moderate-excellent	222 (79.6)	11.85 (2.52)	7.96 (2.10)	3.80 (0.93)
5. Body Mass Index (kg/m^2^)				
< 30	253 (90.7)	11.97 (2.56)	8.16 (2.14)	3.83 (0.93)
≥ 30	26 (9.3)	11.69 (2.65)	8.15 (1.95)	**3.34 (0.74) #**
6. Failed UI surgery ^b^				
No	206 (26.2)	11.67 (2.52)	7.93 (2.06)	3.66 (0.81)
Yes	73 (26.2)	12.71 (2.54) *	8.81 (2.17) *	4.11 (1.14) *
7. Co morbidity (yes) ^c^				
No	206 (73.8)	11.68 (2.59)	7.93 (2.22)	3.71 (0.85)
Yes	73 (26.2)	12.68 (2.37)*	8.81 (1.66) *	**3.96 (1.01) #**
8. Low back pain ^d^				
No	247 (88.5)	11.81 (2.55)	8.05 (2.12)	3.77 (0.93)
Yes	32 (11.5)	**13.00 (2.46) #**	**9.00 (1.93) #**	3.84 (0.88)

^a ^Mixed but dominant stress UI.

^b ^Surgical procedures were related to women's stress urinary incontinence.

^c ^Co morbidity included (n): COPD (31), cardiovascular problems (25), and diabetes type-2 (15).

^d ^(pseudo-) radicular symptoms included (n = 11).

* Significant difference between groups (p < 0.01).

# Significant difference between groups (p < 0.05)

Patients with mixed (but dominant) stress UI or a high BMI (> 30 kg/m^2^) did not show a difference in the total PRAFAB questionnaire or 'leakage severity' scores as hypothesized, but patients with mixed UI experienced a greater symptom impact from their UI compared to those who had not, while patients with a higher BMI experienced a lower impact from their UI. The results of the construct validity analyses also demonstrate the variable relationship between urinary leakage severity and perceived symptom impact subscales.

## Discussion

The primary aim of this study was to investigate the factor structure of the PRAFAB questionnaire and dimensionality of potential subscales in a sample of 279 women with stress UI. The results of our factor analyses demonstrate the more or less expected two-factorial structure of the PRAFAB questionnaire. Therefore, it is strongly recommended to use these two subscales as separate outcomes in future research instead of presenting a total score for the five individual items of the PRAFAB questionnaire. The two factors had adequate to good internal consistency reliability. The inter-subscale correlation was moderate [13-15,19]. Furthermore, the results confirmed the construct validity as demonstrated in our previous psychometric study [7].

To retain items we decided to accept as relevant only factor loadings on individual items of at least 0.40 and to eliminate items from the PRAFAB questionnaire with substantial loadings (> 0.30) on more than one factor [13,15-19]. All the individual items fulfilled these criteria to be retained in the 5-item PRAFAB questionnaire (factor loadings ranged from 0.73 to 0.84 on the individual items) based on the 'simple structure' method but were borderline acceptable for the item adjustment of Factor 2 with a factor loading of 0.40 based on the maximum likelihood analysis (factor loadings ranged from 0.40 to 0.93). The best description of the factorial structure of the PRAFAB questionnaire in this sample of women with stress UI was given by a two-factor solution, measuring stress UI leakage severity (protection, amount and frequency) and perceived symptom impact (body (or self) image but borderline for the item adjustment). The first subscale 'UI leakage severity' may be considered as the strongest factor, with the highest explained variance in all confirmatory factor analyses. Both subscales demonstrated at least adequate internal consistencies (>0.70), inter-item and item-total correlations. The results of our exploratory and confirmatory factor analyses, in both random samples, were very similar with consistent two-factor structure solutions that confirmed the hypothesized and proposed subscales of the PRAFAB questionnaire. As a confirmatory procedure, factor analysis supports the construct validity of the PRAFAB questionnaire measuring stress UI 'leakage severity' and 'perceived symptom impact' of stress UI.

A potential weak point in the analyses could be that the PRAFAB questionnaire item adjustment was skewed, thus not normally distributed. Normality is not a necessary assumption for PCA [13,19], but skewed distributions or outliers could have distorted the results. However, the results of our analyses do not support this potential distortion because the PCA results (e.g. the results of the exploratory factor analysis as well as the results of the 'simple structure' confirmatory factor analysis) were fairly identical and consistent in both study samples, though it might be the reason for the lower and borderline factor loading of the skewed item 'adjustment' as shown by the maximum likelihood analysis. This study confirms the hypothesized and proposed two-factorial structure of the PRAFAB questionnaire using a cross validation study design.

The consequence of the two-factor dimensionality is that this measure is able to make a distinction between different kinds of baseline severity and consequently different kinds of outcomes: 'leakage severity' and 'perceived symptom impact' of stress UI on the patient's life. The correlation between these two scale scores was moderate (r = 0.31) indicating that the scale scores are measuring distinct but related dimensions of the PRAFAB questionnaire and should be used as separate outcomes in future research. The moderate correlation among the subscales is in accordance with what we know from the literature. The perceived symptom impact or consequence of stress UI will be different from patient to patient and not necessarily related to the severity of the stress UI leakage symptoms [2,4,5].

While it is not possible to measure in detail all potential domains of the severity and perceived symptom impact of stress UI, the brief, quick and easy to administer PRAFAB questionnaire covers important aspects of stress UI, with hardly any burden for the practitioners or the patient, leading us to recommend its use in daily clinical practice. However, we believe that in clinical practice it is still worthwhile examining the individual PRAFAB questionnaire items for a better understanding of stress UI leakage severity, perceived symptom impact, goals and type of physiotherapy or other interventions for individual patients.

Looking at the PRAFAB questionnaire in more detail from the biopsychosocial perspective, the PRAFAB questionnaire could have potentially more than two meaningful factors. For example, the amount and frequency items might represent biological (impairment) factors, the body (or self) image item the 'psychological' factor of bother and the item adjustment (participation restrictions) the 'social' factor. In other words this instrument might also fit the biopsychosocial approach. From our point of view the item 'protection' was not assumed to be included in the UI leakage severity subscale (Factor 1) but, although strongly related to the amount and frequency of urine loss, we considered 'protection' as a consequence or anticipation of a likely event of urine loss. Despite that, the single item 'protection' is informative and interesting from the perspective of self-confidence for individual women or from the perspective of cost to society. For example, the costs of incontinence absorbent materials in the Netherlands were estimated at 119.3 million euro in 2004 [1,2]. Despite the impact of UI on quality of life and cost to society only a minority who had consulted their GP were receiving treatment, though some could have benefited from an effective intervention [1,2]. Figures in the Netherlands showed that of all women who had consulted their GP only 1.9% had been referred to urogynaecologists and 1.6% to specialized (and registered) physiotherapists in women's health [2]. GPs and other (primary) care professionals need to be more alert in early identification and referral of incontinent patients, offering tailored interventions as recently stated in Dutch clinical urinary incontinence guidelines as a vehicle for integrated (primary health) care [27].

The patterns of the PRAFAB questionnaire scores in relation to the baseline characteristics also support the construct validity of the PRAFAB questionnaire scores in this study and are consistent with our expectations as demonstrated in our previous psychometric study [7] and for example by Fultz et al. [4] and Melville et al. [6]. As expected, the total PRAFAB questionnaire scores and urinary leakage severity scores were higher for patients referred by urogynaecologists compared with those referred by GPs, for patients with a self-rated lower physical health, failed UI-related surgery, co-morbid conditions and chronic low back pain. Patients with a higher education (compared to those who had not) had lower leakage severity and perceived symptom impact scores. These confirmative findings underscore the validity of the PRAFAB questionnaire. Furthermore it shows that patients with higher scores on the subscale 'leakage severity' often perceived a stronger impact of UI but this seems to be more variable.

Temporary or permanent stress UI symptoms affect activities in daily life and may have a firm psychosocial impact [2,4-6] that also needs to be addressed by the health professionals involved. Brief, valid and responsive outcome measures that can be used easily in clinical practice are therefore highly recommended. For example, other and competing outcome measures such as the Incontinence Consultation on Incontinence Questionnaire (ICIQ) [28] or the Incontinence Severity Index (ISI) [29,30], which also demonstrate the face-validity of the PRAFAB questionnaire based on comparable items. However, the ISI only focuses on urinary leakage severity and not on the perceived impact of UI. The availability of brief and simple outcome measures, which lessen the scoring burden for practitioners, will probably enhance the implementation of these outcome measures in clinical practice and will also enhance informed decision-making in open communication with the patient.

## Conclusion

In summary, the findings demonstrate high factorial validity and construct validity of the PRAFAB questionnaire and subscales. The subscales should be used as separate outcomes in future research and clinical practice. However, the generalization of the two-factor solution for different types of UI, higher age groups or for example to a male population is still unclear. Further studies should examine whether the questionnaire is also valid in other (sub) populations. Nevertheless the results are promising.

## Competing interests

The author(s) declare that they have no competing interests.

## Authors' contributions

EH obtained funding, had full access to all of the data in the study and takes responsibility for the integrity of the data and accuracy of the data analysis. EH and AB were responsible for the acquisition of data. EH, RB and HdV set up the design of the study. EH, AB, BS and HdV were responsible for the analysis and interpretation. EH, AB, BS, RB and HdV critically contributed to the manuscript and all of the revisions of the final manuscript for important intellectual content. All authors read and approved the final manuscript.

## Pre-publication history

The pre-publication history for this paper can be accessed here:


